# Lack of Serologic Evidence of *Neospora caninum* in Humans, England

**DOI:** 10.3201/eid1406.071128

**Published:** 2008-06

**Authors:** Catherine M. McCann, Andrew J. Vyse, Roland L Salmon, Daniel Thomas, Diana J.L. Williams, John W. McGarry, Richard Pebody, Alexander J. Trees

**Affiliations:** * University of Liverpool, Liverpool, UK; † Health Protection Agency, London, UK; ‡ National Public Health Service for Wales, Cardiff, Wales, UK

**Keywords:** Neospora caninum, zoonosis, human, serology, dispatch

## Abstract

Retrospective testing of 3,232 serum samples from the general population and 518 serum samples from a high-risk group showed no evidence of human exposure to *Neospora caninum* in England. Results were obtained by using immunofluorescence antibody testing and ELISA to analyze frequency distribution.

The protozoan parasite *Neospora caninum* has recently emerged as a major cause of disease in cattle and dogs worldwide (*1*). An issue of concern is that *N. caninum* might be zoonotic because of its close biologic relationship to the common zoonotic parasite *Toxoplasma gondii* and because rhesus monkeys have been experimentally infected (*2*). Humans could become exposed to *N. caninum* by accidental ingestion of oocysts shed in the feces of canid definitive hosts or following the consumption of raw or inadequately cooked meat that contains tissue cysts. This retrospective study sought immunologic evidence of human exposure to *N. caninum* in England. Two cohorts of the population were examined: a convenience collection, which approximated the general population, and a putative high-risk group.

## The Study

The first cohort comprised anonymized residues of serum samples submitted in 2000 for microbiologic or biochemical testing for diagnostic or screening purposes to 11 laboratories in England that were contributing to the Public Health Laboratories Service (PHLS) Serologic Surveillance Programme (now Health Protection Agency [HPA]) Seroepidemiology Programme (*3*). The second cohort comprised 518 samples collected in 1995 from the PHLS cohort of farm workers, which was recruited in 1991 to provide annual samples from a population at high risk for zoonotic infections from livestock (*4*,*5*). Ethical approval was granted by relevant ethics committees.

Serum specimens were initially screened at a dilution of 1:10 in phosphate-buffered saline (PBS), pH 7.2, containing Tween 20 (PBS/Tween) with an inhibition ELISA developed in our laboratory and previously validated in cattle and dogs (*6*). Positive and negative bovine serum controls were used on each ELISA plate. In the absence of a human positive control, we used a primate serum sample as a further positive control. Optical density was read at 450 nm, and percentage inhibition (PI) values were calculated by using the formula 100 – [(test OD/negative control OD) ×100]. Without specific validation, a cut-off of 20% inhibition was chosen to indicate putative positives. This cut-off has been used for previous comparison of the inhibition ELISA with a conventional *N. caninum*–specific ELISA in bovine sera (*6*). All samples with inhibition >20% were subsequently tested in an immunofluorescence test (IFAT) (*7*) with appropriate species-specific fluorescein isothiocynate conjugates. All samples were tested at a dilution of 1:50 with positive controls of bovine and primate *N. caninum* serum and bovine negative control serum as above. For the inhibition ELISA results, the distribution of the data was examined first by plotting percent inhibition, with the data aggregated into bands of 10%. The plots were repeated after logging the data and putting it into bins (equal width reactivity categories based on log 10% [log_10_] inhibition). The distribution of log_10_ inhibition was also examined according to person’s sex and age and the submitting laboratory.

From the HPA collection, 3,232 samples from persons 20–70 years of age were tested; 1,889 (58.45%) were from women. The PHLS farm cohort, comprised 74% men and 26% women, with a median age range of 41–50 years (*5*). Six hundred and ninety-one (21.38%) of the HPA samples and 29 (5.56%) of the PHLS samples produced percentage inhibition of >20% in the inhibition ELISA test (Table). For the bovine-positive controls, mean percentage inhibition was 79.76% (5% confidence interval [CI] 78.94–80.58, n = 200). The primate-positive serum had a PI of 63.00% (average of 2 tests). When samples with a PI >20% in the inhibition ELISA were tested by IFAT, all failed to give positive fluorescence results, whereas the primate- and bovine-positive controls consistently gave positive fluorescence results.

**Table:** Results of inhibition ELISA for *Neospora caninum*, England*

Population	% inhibition					No. samples tested
	<20	>20–<30	>30–<40	>40–<50	>50	
HPA seroepidemiology study	2,541	350	201	98	42	3,232
PHLS farm workers cohort	499	12	14	3	0	518

*HPA, Health Protection Agency; PHLS, Public Health Laboratory Service.

The frequency distribution of actual percentage inhibitions for the HPA samples showed a single positively skewed distribution. After the data were logged, the plot showed a single log-normal distribution, with a mean close to 0% inhibition, i.e., similar to the negative control used. There was no evidence that the percentage inhibition differed by sex or region (results not shown) or by age (Figure). The log-transformed inhibition data for the PHLS cohort also produced a single normal frequency distribution about a mean close to 0% inhibition (Figure).

**Figure:**
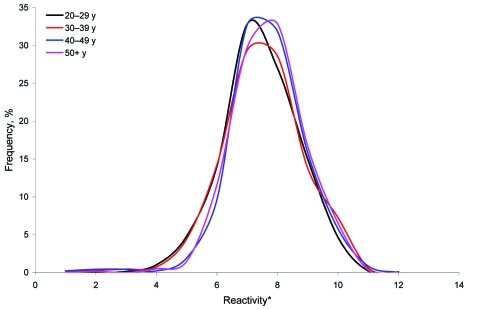
Frequency distribution of inhibition ELISA results for *Neospora caninum*, England (Health Protection Agency serum samples), stratified by age group. *Equal-width bands based on log_10_ percentage inhibition.

In this study, we sought evidence of human exposure to *N. caninum* infection by specific antibody detection in 2 populations. Since no serologic assay has been validated for *N. caninum* antibodies in humans, we used an inhibition ELISA and analyzed the frequency distribution of the percentage inhibitions. Although initial screening gave several putative positives, when the frequency distribution curves were plotted, no evidence was found showing that the samples were distributed discretely into those with antibody to *Neospora* spp. and those without. This provides strong evidence that no *Neospora*-specific immunoglobulin G was found in any of the samples and that the distribution observed describes a single population of nonexposed persons. Moreover, no change in distribution was observed with age, further evidence that the dataset is from a single large population for which results were negative. Finally, confirmatory testing with the IFAT of all samples with >20% inhibition in the inhibition ELISA, failed to detect any specific fluorescence indicative of true positives. The fact that the predictive value of a negative test is very high in low prevalence populations, even with tests of modest performance, further supports the conclusion that infection with *N. caninum* is unlikely. We therefore conclude that these sera show no evidence of exposure to *N. caninum*.

Selection bias in the HPA sera collection is unlikely because of the provision of free access to healthcare for all by the National Health Service. Moreover, a convenience collection has been shown to be comparable to a randomized sample in previous studies of population immunity (*8*). The collection may therefore be considered to approximate the general population of England in terms of its exposure to *N. caninum*. In contrast, the PHLS farm cohort may be regarded as a relatively high-risk group for *N. caninum* infection since those persons would be predicted to have greater than normal exposure to bovine placentas, fetal membranes, and fluids that are potentially infected, or to environments contaminated by feces of dogs, which have access to tissues from potentially infected cattle.

Analyses to detect parasites or parasite DNA have shown no definitive evidence of human infection with *N. caninum* (*1*), but seropositivity has been reported in 3 studies (*9*–*11*) although not in 2 other European surveys (*12*,*13*). Of the 3 reports describing seropositivity, 2 (*10*,*11*) noted a high correlation with seropositivity to *T. gondii*, and, in all, antibody titers versus *N. caninum* antigen were low (at <1:100 dilution). The interpretation of serologic results is difficult, and this study’s approach, in which large numbers of samples were quantitatively assayed to give a frequency distribution, may provide a helpful means of addressing this uncertainty.

## Conclusions

No evidence of human exposure to *N. caninum* was found in a high-risk population in England sampled in 1995 and in a sample of the general population of England collected in 2000. These results suggest that human infection is unlikely in England. However, given global variation in infection prevalence in cattle and possible regional differences in the incidence of oocyst shedding by dogs, there remains a need worldwide to remain vigilant to the possibility of human infection.
